# Immature mice are more susceptible than adult mice to acetaminophen-induced acute liver injury

**DOI:** 10.1038/srep42736

**Published:** 2017-02-16

**Authors:** Yan Lu, Cheng Zhang, Yuan-Hua Chen, Hua Wang, Zhi-Hui Zhang, Xi Chen, De-Xiang Xu

**Affiliations:** 1 Department of Toxicology, Anhui Medical University, Hefei, 230032, China; 2 Anhui Provincial Key Laboratory of Population Health & Aristogenics, Anhui Medical University, Hefei, 230032, China; 3 Second Affiliated Hospital, Anhui Medical University, Hefei 230601, China; 4 First Affiliated Hospital, Anhui Medical University, Hefei 230022, China

## Abstract

Acetaminophen (APAP) overdose induces acute liver injury. The aim of the present study was to analyze the difference of susceptibility between immature and adult mice to APAP-induced acute liver injury. Weanling immature and adult mice were injected with APAP (300 mg/kg). As expected, immature mice were more susceptible than adult mice to APAP-induced acute liver injury. APAP-evoked hepatic c-Jun N-terminal kinase phosphorylation was stronger in immature mice than in adult mice. Hepatic receptor-interacting protein (RIP)1 was obviously activated at APAP-exposed immature and adult mice. Interestingly, hepatic RIP3 activation was more obvious in APAP-treated immature mice than adult mice. Although there was no difference on hepatic GSH metabolic enzymes between immature and adult mice, immature mice were more susceptible than adult mice to APAP-induced hepatic GSH depletion. Of interest, immature mice expressed a much higher level of hepatic *Cyp2e1* and *Cyp3a11* mRNAs than adult mice. Correspondingly, immature mice expressed a higher level of hepatic CYP2E1, the key drug metabolic enzyme that metabolized APAP into the reactive metabolite NAPQI. These results suggest that a higher level of hepatic drug metabolic enzymes in immature mice than adult mice might contribute to the difference of susceptibility to APAP-induced acute liver injury.

Acetaminophen (APAP) is a widely used analgesic and antipyretic drug. Although it is safe at therapeutic doses, APAP overdose induces acute liver injury^1^,^2^,^3^. APAP-induced acute liver injury is initiated by the reactive metabolite, N-acetyl-p-benzoquinone imine (NAPQI), which is generated by several cytochrome P450 (CYP) isoenzymes, mainly CYP2E1 and CYP3A4^4^,^5^,^6^,^7^,^8^,^9^,^10^. Several studies demonstrate that the prolonged activation of hepatic c-Jun N-terminal kinase (JNK) is involved in APAP-induced hepatocyte death^11^,^12^. Moreover, apoptosis-inducing factor (AIF) is also a critical mediator of hepatocyte death during APAP-evoked acute liver injury^13^,^14^. Recently, several studies demonstrate that hepatic receptor interacting protein (RIP)1 and RIP3 activation is involved in hepatocyte death during APAP-induced acute liver injury^15^,^16^,^17^,^18^.

APAP is one of the most popular drugs for antipyretic and analgesic treatment in pediatric patients^19^. According to several epidemiological investigations, APAP-induced hepatotoxicity is the most common identifiable cause of acute liver failure in children^20^,^21^,^22^,^23^,^24^. On the other hand, a recent study showed that old male Fischer 344 rats were less susceptible than younger rats to APAP-induced acute liver injury^25^, indicating that there might be differences of the susceptibility between young and old patients to APAP-induced acute liver injury. Nevertheless, whether there are also differences of the susceptibility between young children and adults to APAP-induced acute liver injury remains to be determined.

The aim of the present study was to analyze the difference of the susceptibility between weanling immature mice and adult mice to APAP-induced acute liver injury. Our results showed that immature mice were more susceptible than adult mice to APAP-induced acute liver injury. We found that immature mice were more susceptible than adult mice to APAP-evoked hepatic GSH depletion. We demonstrate for the first time that a higher level of hepatic drug metabolic enzymes in immature mice than adult mice might partially contribute to the difference of the susceptibility to APAP-induced acute liver injury.

## Results

### Immature mice are more susceptible than adult mice to APAP-induced acute liver injury

**Figure 1:**
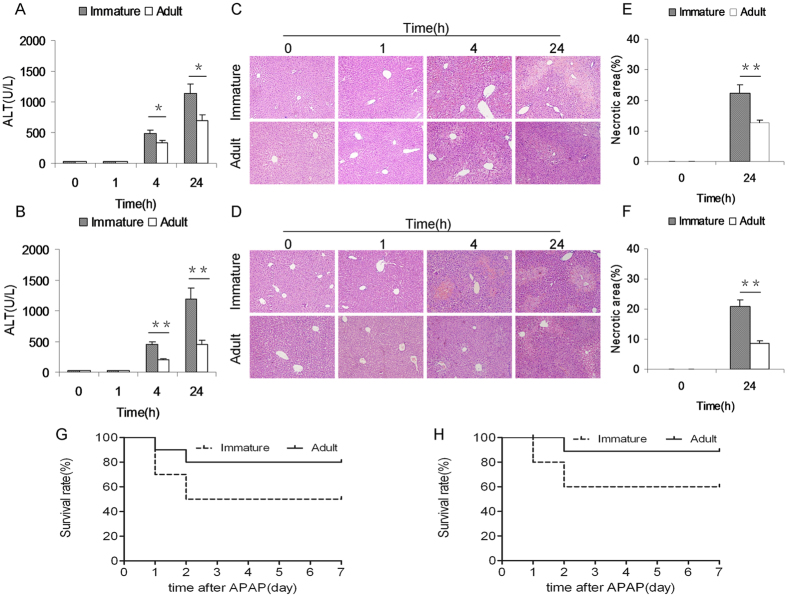
Immature mice are more susceptible than adult mice to APAP-induced acute liver injury. Weanling and adult mice were i.p. injected with APAP (300 mg/kg). (**A**: Male and **B**: Female) Sera was collected at different time points (0, 1, 4 and 24 h) after APAP. Serum ALT was analyzed between immature mice and adult mice. (**C**: Male and **D**: Female) Representative photomicrographs of liver histology (**H** & **E,** magnification: 100×). (**E**: Male and **F**: Female) The percentage of necrotic area was analyzed. (**G**: Male and **H**: Female) Forty mice (10 adult males, 10 weanling males, 10 adult females and 10 weanling females) were i.p. injected with APAP (300 mg/kg). Animal death was observed until 72 h after APAP. All data were expressed as means ± SE (n = 10). *P < 0.05, **P < 0.01.

APAP-induced acute liver injury was compared between immature and adult mice. As expected, serum ALT was significantly elevated 4 h after APAP and remaining increased 24 h after APAP (Fig. 1A,B). Further analysis showed that serum ALT in APAP-treated immature males was higher than that of adult males (Fig. 1A). Similarly, serum ALT in APAP-treated immature females was higher than that of adult females (Fig. 1B). The relative liver weight (liver weight/body weight) was compared between immature and adult mice. As expected, the relative liver weight was elevated 4 h after APAP (data not shown). Further analysis showed that the relative liver weight in APAP-treated immature males was higher than that of adult males (data not shown). Similarly, the relative liver weight in APAP-treated immature females was higher than that of adult females (data not shown). Histopathology showed a characteristic centrilobular necrosis 4 h and 24 h after APAP (Fig. 1C,D). Further analysis showed that necrotic area in APAP-treated immature males was more than that of adult males (Fig. 1E). Similarly, necrotic area in APAP-treated immature females was more than that of adult females (Fig. 1F). Survival test showed that only 20% (2/10) of adult males and 10% (1/10) of adult females were dead until 72 h after APAP (Fig. 1G,H). Of interest, 50% (5/10) of immature males and 40% (4/10) of immature females were dead until 72 h after APAP (Fig. 1G,H). The difference of the susceptibility between male and female mice to APAP-induced acute liver injury was analyzed. As shown in Supplementary Fig S1, only adult male mice are more susceptible than adult female mice to APAP-induced acute liver injury. There was no significant difference on serum ALT and hepatic histopathologic damage between APAP-treated immature male and female mice (Supplementary Fig. S1).

### Immature mice are more susceptible than adult mice to APAP-induced hepatocyte death

**Figure 2:**
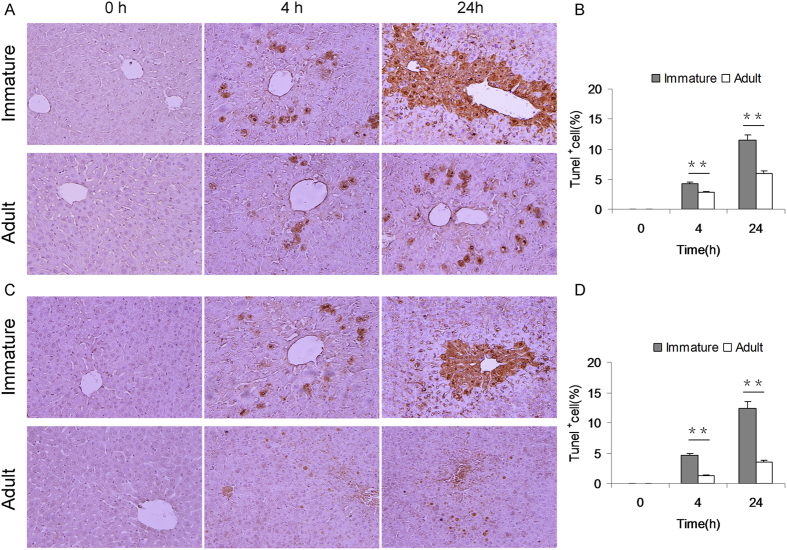
Immature mice are more susceptible than adult mice to APAP-induced hepatocyte death. Weanling and adult mice were i.p. injected with APAP (300 mg/kg). Liver samples were collected at 4 and 24 h after APAP. Hepatocyte death was determined using TUNEL assay. (**A**) Representative photomicrographs from male mice. (**B**) The number of TUNEL + hepatocytes was compared between immature and adult male mice. (**C**) Representative photomicrographs from female mice. (**D**) The number of TUNEL + hepatocytes was compared between immature and adult female mice. All data were expressed as means ± SE (n = 10). **P < 0.01.

APAP-induced hepatocyte death was determined by TUNEL assay. As expected, numerous TUNEL+ hepatocytes were observed at 4 h and 24 h after APAP (Fig. 2A,C). Further analysis showed that the number of TUNEL+ hepatocytes in APAP-treated immature males was more than that of adult males (Fig. 2B). Similarly, the number of TUNEL+ hepatocytes in APAP-treated immature females was more than that of adult females (Fig. 2D).

### APAP-induced hepatic JNK phosphorylation is stronger in immature mice than adult mice

**Figure 3:**
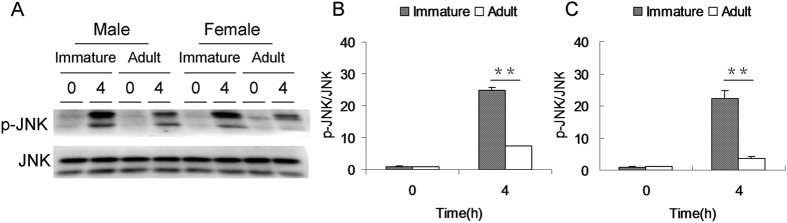
APAP-induced hepatic JNK phosphorylation is stronger in immature mice than adult mice. Weanling and adult mice were i.p. injected with APAP (300 mg/kg). Liver samples were collected 4 h after APAP. Hepatic JNK phosphorylation was detected by immunoblot. (**A**) A representative gel for pJNK (upper panel) and JNK (lower panel) was shown. (**B**) Quantitative analysis of scanning densitometry was performed between immature and adult male mice. (**C**) Quantitative analysis of scanning densitometry was performed between immature and adult female mice. All data were expressed as means ± SE (n = 10) from three independent experiments. **P < 0.01.

APAP-induced hepatic JNK phosphorylation was compared in immature and adult mice. As expected, the level of hepatic pJNK was significantly elevated at 4 h after APAP (Fig. 3A). Further analysis showed that the level of hepatic pJNK in APAP-treated immature male mice was higher than that of adult male mice (Fig. 3B). Similarly, the level of hepatic pJNK in APAP-treated immature female mice was higher than that of adult female mice (Fig. 3C).

### APAP differentially regulates hepatic RIP1 and RIP3 in immature and adult mice

**Figure 4:**
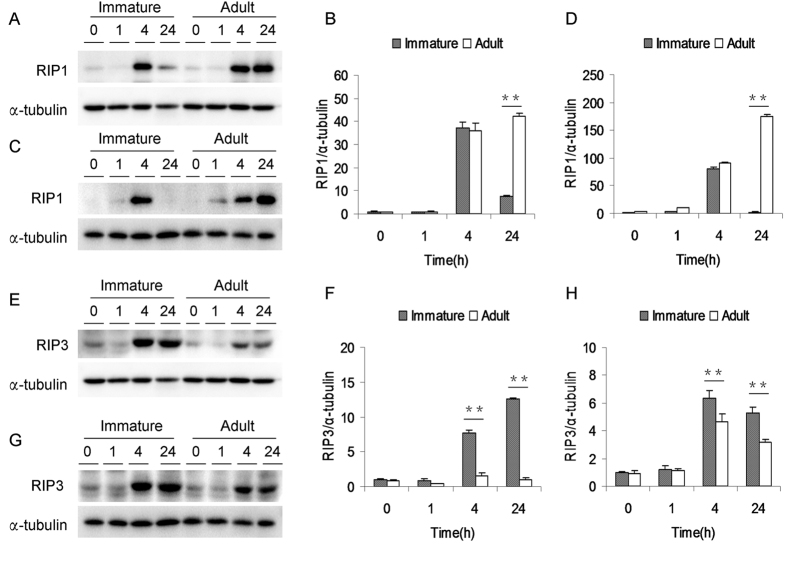
APAP differentially induces hepatic RIP1 and RIP3 in immature and adult mice. Weanling and adult mice were i.p. injected with APAP (300 mg/kg). Liver samples were collected at different time points (0, 1, 4 and 24 h) after APAP. (**A–D**) Hepatic RIP1 was detected by immunoblot. (**A**) A representative gel for RIP1 (upper panel) and α-tubulin (lower panel) from immature and adult male mice was shown. (**B**) Quantitative analysis of scanning densitometry was performed between immature and adult male mice. (**C**) A representative gel for RIP1 (upper panel) and α-tubulin (lower panel) from immature and adult female mice was shown. (**D**) Quantitative analysis of scanning densitometry was performed between immature and adult female mice. (**E–H**) Hepatic RIP3 was detected by immunoblot. (**E**) A representative gel for RIP3 (upper panel) and α-tubulin (lower panel) from immature and adult male mice was shown. (**F**) Quantitative analysis of scanning densitometry was performed between immature and adult male mice. (**G**) A representative gel for RIP3 (upper panel) and α-tubulin (lower panel) from immature and adult female mice was shown. (**H**) Quantitative analysis of scanning densitometry was performed between immature and adult female mice. All data were expressed as means ± SE (n = 10) from three independent experiments. **P < 0.01.

APAP-induced hepatic RIP1 were analyzed in immature and adult mice. As shown in Fig. 4A,B, hepatic RIP1 levels in immature and adult male mice were elevated at 4 h after APAP. Although hepatic RIP1 in immature male mice dropped to a low level, hepatic RIP1 level in adult male mice remained elevated at 24 h after APAP. Similarly, hepatic RIP1 levels in immature and adult female mice were significantly elevated at 4 h after APAP. Although hepatic RIP1 in immature female mice dropped to normal level, but hepatic RIP1 level in adult female mice remained elevated at 24 h after APAP (Fig. 4C,D). APAP-induced hepatic RIP3 was then analyzed in immature and adult mice. As shown in Fig. 4E,F, hepatic RIP3 level in male mice was elevated at 4 h after APAP and remaining elevated at 24 h after APAP. Of interest, hepatic RIP3 level in APAP-treated immature male mice was higher than that of adult male mice. Similarly, hepatic RIP3 level in female mice was elevated at 4 h after APAP and remaining elevated at 24 h after APAP (Fig. 4G). Moreover, hepatic RIP3 level in APAP-treated immature female mice was higher than that of adult female mice (Fig. 4H).

### Immature mice are more susceptible than adult mice to APAP-induced hepatic GSH depletion

**Figure 5:**
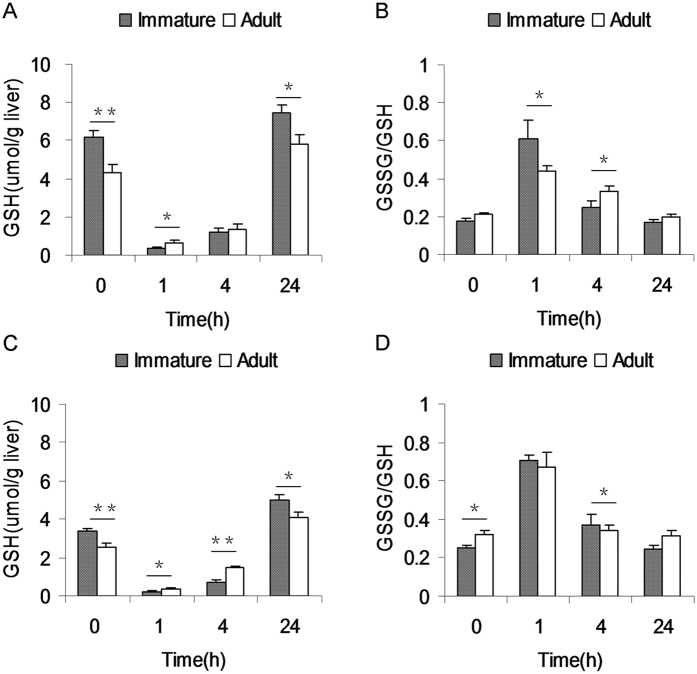
Immature mice are more susceptible than adult mice to APAP-induced hepatic GSH depletion. Weanling and adult mice were i.p. injected with APAP (300 mg/kg). Liver samples were collected at different time points (0, 1, 4 and 24 h) after APAP. Hepatic GSH and GSSG contents were measured. (**A**) Hepatic GSH content was compared between immature and adult male mice. (**B**) Hepatic GSH/GSSG was compared between immature and adult male mice. (**C**) Hepatic GSH content was analyzed between immature and adult female mice. (**D**) Hepatic GSH/GSSG was compared between immature and adult female mice. All data were expressed as means ± SE (n = 10). *P < 0.05, **P < 0.01.

As shown in Fig. 5A, hepatic GSH content in APAP-untreated immature male mice was higher than that of APAP-untreated adult male mice. Similarly, hepatic GSH content in APAP-untreated immature female mice was higher than that of APAP-untreated adult female mice (Fig. 5C). APAP-evoked hepatic GSH depletion was analyzed. As expected, hepatic GSH was depleted at 1 h after APAP and remained significantly reduced at 4 h after APAP (Fig. 5A,C). APAP-evoked hepatic GSH depletion was compared between immature and adult male mice. As shown in Fig. 5A, hepatic GSH content at 1 h after APAP were lower in immature males than that of adult males. By contrary, GSSG/GSH at 1 h after APAP was higher in immature males than that of adult males (Fig. 5B). APAP-evoked hepatic GSH depletion was then compared between immature and adult female mice. As shown in Fig. 5C, hepatic GSH contents at 1 and 4 h after APAP were lower in immature females than that of adult females. No significant difference on GSSG/GSH at 1 h after APAP between immature and adult females, but GSSG/GSH at 4 h after APAP was higher in immature females than that of adult females (Fig. 5D).

### Immature mice do not exhibit less hepatic GSH metabolic activities

**Figure 6:**
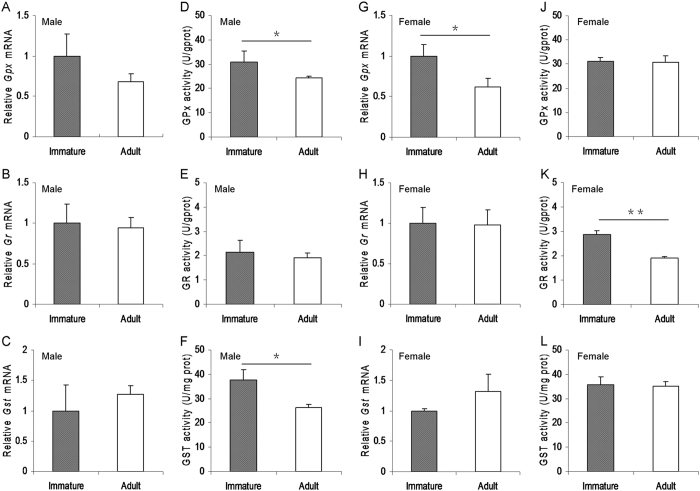
Immature mice do not exhibit less hepatic GSH metabolic activities. Weanling and adult mice were i.p. injected with APAP (300 mg/kg). Liver samples were collected from APAP-untreated immature and adult mice. (**A–C**) Hepatic GSH metabolic enzyme genes in immature and adult male mice were measured using real-time RT-PCR. (**D–F**) Hepatic GSH metabolic enzyme activities in immature and adult male mice were measured. (**G–I**) Hepatic GSH metabolic enzyme genes in immature and adult female mice were measured using real-time RT-PCR. (**J–L**) Hepatic GSH metabolic enzyme activities in immature and adult female mice were measured. All data were expressed as means ± SE (n = 10) from three independent experiments. *P < 0.05, **P < 0.01.

To investigate whether immature mice exhibit less hepatic GSH metabolic activities, the expression of hepatic GSH metabolic enzymes and their metabolic activities were analyzed between immature and adult male mice. As shown in Fig. 6A–C, there was no significant difference on the expression of *hepatic glutathione peroxidase (Gpx), glutathione reductase (Gr)* and *glutathione S-transferases (Gst)*, three GSH metabolic enzyme genes, between immature and adult male mice. Actually, hepatic GPX and GST activities in immature male mice were slightly higher than that of adult male mice (Fig. 6D–F). The expression of hepatic GSH metabolic enzyme genes was then analyzed between immature and adult female mice. As expected, no significant difference on the expression of hepatic *Gr* and *Gst* was observed between immature and adult female mice (Fig. 6H,I). Actually, hepatic *Gpx* mRNA level in immature female mice was slightly higher than that of adult female mice (Fig. 6G). Moreover, there was no significant difference on hepatic GPX and GST metabolic activities between immature and adult female mice (Fig. 6J,L). Actually, hepatic GR activity in immature female mice was slightly higher than that of adult female mice (Fig. 6K).

### Immature mice express a higher level of hepatic drug metabolic enzymes than adult mice

**Figure 7:**
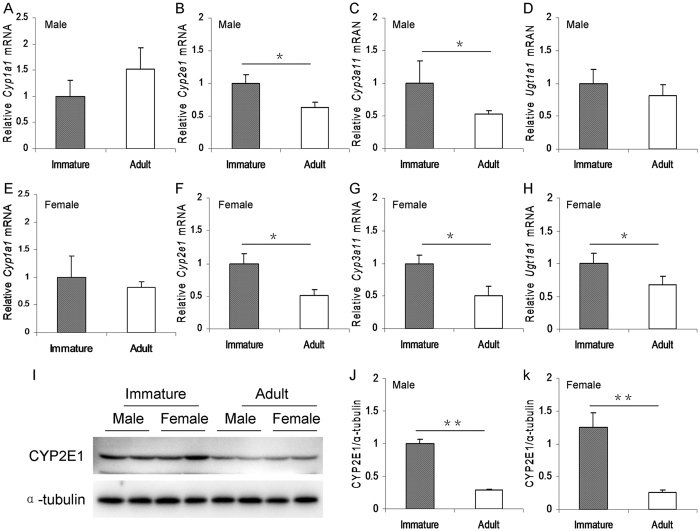
Immature mice express a higher level of hepatic drug metabolic enzymes than adult mice. Weanling and adult mice were i.p. injected with APAP (300 mg/kg). Liver samples were collected from APAP-untreated immature and adult mice. (**A–D**) Hepatic drug metabolic enzyme genes (**A**, *Cyp1a1*; **B**, *Cyp2e1*; C, *Cyp3a11*; **D**, *Ugt1a1*) in immature and adult male mice were measured using real-time RT-PCR. (**E–H**) Hepatic drug metabolic enzyme genes (**E**, *Cyp1a1*; **F**, *Cyp2e1*; **G**, *Cyp3a11*; **H**, *Ugt1a1*) in immature and adult female mice were measured using real-time RT-PCR. (**I–K**) Hepatic CYP2E1 in immature and adult mice was detected by immunoblot. (**I**) A representative gel for CYP2E1 (upper panel) and α-tubulin (lower panel) from immature and adult mice was shown. (**J**) Quantitative analysis of scanning densitometry was performed between immature and adult male mice. (**K**) Quantitative analysis of scanning densitometry was performed between immature and adult female mice. All data were expressed as means ± SE (n = 10) from three independent experiments. *P < 0.05, **P < 0.01.

The difference on the expression of hepatic *Cyp1a1, Cyp2e1, Cyp3a11* and *Ugt1a1*, four key metabolic enzyme genes for APAP, was compared between immature and adult male mice. Although no difference on hepatic *Cyp1a1* and *Ugt1a1* mRNAs was observed between immature and adult male mice (Fig. 7A,D), hepatic *Cyp2e1* and *Cyp3a11* mRNAs in immature males were higher than that of adult males (Fig. 7B,C). Similarly, hepatic *Cyp2e1, Cyp3a11* and *Ugt1a1* mRNAs in immature females were higher than that of adult females (Fig. 7F–H). There was no significant difference on the expression of hepatic *Cyp1a1* between immature and adult females (Fig. 7E). The difference on hepatic CYP2E1 protein, the key metabolic enzyme for APAP, was then analyzed between immature and adult mice. Hepatic CYP2E1 level in immature males was higher than that of adult males (Fig. 7I,J). Similarly, hepatic CYP2E1 level in immature females was higher than that of adult females (Fig. 7I,K).

## Discussion

The present study investigated differential susceptibility between immature and adult mice to APAP-induced acute liver injury. We showed that serum ALT levels in APAP-treated immature male and female mice were higher than that of adult male and female mice. Moreover, the numbers of TUNEL+ hepatocytes in APAP-treated immature male and female mice were more than that of adult male and female mice. In addition, the necrotic areas in APAP-treated immature male and female mice were more than that of adult male and female mice. In contrast, the survival rates in APAP-treated immature male and female mice were lower than that of adult male and female mice. These results suggest that immature mice are more susceptible than adult mice to APAP-induced acute liver injury.

Several studies demonstrated that the sustained activation of hepatic JNK was involved in APAP-induced acute liver injury^11^,^12^,^26^. The role of RIP1 and RIP3-mediated necroptosis in APAP-induced liver injury remains controversial^27^. Several reports showed that hepatic RIP1 activation played an important role in APAP-induced hepatocyte death^15^,^16^,^17^. In addition, RIP3 was also a critical early mediator of APAP-induced hepatocyte necroptosis^18^. However, a recent report found that RIP1 in hepatocytes did not mediate APAP-evoked hepatotoxicity in conditional knockout mice lacking RIPK1 only in liver parenchymal cells^28^. In the present study, we showed that hepatic JNK was rapidly phosphorylated in APAP-treated immature mice and adult mice. At the same time, hepatic RIP1 and RIP3 were activated in APAP-treated immature mice and adult mice. Of interest, hepatic JNK phosphorylation was stronger in APAP-treated immature mice than in adult mice. In addition, hepatic RIP3 activation was more obvious in APAP-treated immature mice than in adult mice. These results suggest that APAP-activated hepatic JNK and RIP signaling are stronger in immature mice than in adult mice.

GSH is an important cellular antioxidant in the liver. Increasing evidence demonstrates that hepatic GSH depletion is an important event at the early stage of APAP-induced acute liver injury^29^,^30^,^31^. Indeed, the present study showed that hepatic GSH was almost completely depleted as early as 1 h after APAP injection. Although hepatic GSH content before APAP injection was higher in immature mice than in adult mice, hepatic GSH content 1 h after APAP was much lower in immature male mice than in adult male mice. Similarly, hepatic GSH content 1 h after APAP was much lower in immature female mice than in adult female mice. These results suggest that immature mice are more susceptible than adult mice to APAP-induced hepatic GSH depletion. Cellular GSH content is usually regulated by several GSH metabolic enzymes involved in GSH synthesis, such as GSH synthetase and γ-glutamylcysteine synthetase, GSH recycling, mainly GR, and GSH consumption, mainly GST and GPX^32^. In the present study, we analyzed the difference of several GSH metabolic enzymes between immature mice and adult mice. We found that there was no significant difference on the expression of hepatic GSH metabolic enzyme genes. Actually, hepatic GPX and GST activities in immature male mice and hepatic GR activity in immature female mice were slightly higher than that of adult mice. These results suggest that the differential susceptibility between immature mice and adult mice to APAP-induced hepatic GSH depletion cannot be attributed to the difference of hepatic GSH metabolism.

Hepatic metabolic biotransformation into the reactive metabolite NAPQI mediated by several drug metabolic enzymes, mainly CYP2E1 and CYP3A, is essential for APAP-induced hepatic GSH depletion and subsequent acute liver injury^33^. Several early reports showed that inhibition of CYP2E1 significantly reduced the formation of APAP-oxidized metabolites and effectually protected mice from APAP-induced acute liver injury^34^,^35^,^36^. On the contrary, alcohol-induced up-regulation of hepatic CYP2E1 and CYP3A elevated the formation of APAP-oxidized metabolites and exacerbated APAP-induced acute liver injury^37^,^38^,^39^. In the present study, we analyzed the difference on the expression of hepatic drug metabolic enzymes between immature and adult mice. Although no significant difference on hepatic *Cyp1a1* and *Ugt1a1* expression was observed between immature and adult mice, hepatic *Cyp2e1* and *Cyp3a11* mRNAs in immature male mice were higher than that of adult male mice. Similarly, hepatic *Cyp2e1, Cyp3a11* and *Ugt1a1* mRNAs were higher in immature female mice than in adult female mice. Moreover, the levels of hepatic CYP2E1 protein were higher in immature male and female mice than in adult male and female mice. Therefore, we speculate that a higher level of hepatic drug metabolic enzymes in immature mice, which metabolizes APAP into more reactive metabolite NAPQI, might contribute, at least partially, to more susceptible than adult mice to APAP-induced acute liver injury.

This study laid emphasis on the difference of the susceptibility between immature and adult mice to APAP-induced acute liver injury. A recent report showed that male mice were more susceptible than female mice to APAP-induced acute liver injury^40^. The present study analyzed the difference of the susceptibility between male and female mice to APAP-induced acute liver injury. We showed that only adult male mice are more susceptible than adult female mice to APAP-induced acute liver injury. Unexpectedly, there was no difference of the susceptibility between immature males and females to APAP-induced acute liver injury. Thus, additional work is necessary to determine the reason that there is a gender difference of the susceptibility in adult mice but not in immature mice. The present study has several limitations. First, the present study did not measure hepatic CYP2E1 and CYP3A activities in immature and adult mice. Second, the present study did not analyze the difference of the reactive metabolite NAPQI between APAP-treated immature and adult mice. Thus, additional work is required to analyze hepatic CYP2E1 and CYP3A activities in immature and adult mice. In addition, the clinical relevance of these animal findings needs to be determined in the future research.

In summary, this study analyzed the difference of susceptibility between immature mice and adult mice to APAP-induced acute liver injury. Our results showed that hepatic RIP1 was obviously activated in APAP-exposed immature and adult mice. Of interest, APAP-evoked hepatic JNK phosphorylation was stronger in immature mice than in adult mice. Moreover, Hepatic RIP3 activation was more obvious in APAP-treated immature mice than adult mice. We found that immature mice were more susceptible than adult mice to APAP-evoked hepatic GSH depletion and acute liver injury. We demonstrate that the difference of hepatic drug metabolic enzymes between immature mice and adult mice might partially contribute to the differential susceptibility to APAP-induced acute liver injury.

## Materials and Methods

### Chemicals and reagents

Acetaminophen (APAP) was purchased from Sigma Chemical Co. (St. Louis, MO). RIP1, JNK and pJNK antibodies were from Cell Signaling. RIP3 antibody was from ProSci Incorporated (San Diego, California, USA). Alpha-tubulin antibody was from Santa Cruz Biotechnologies (Santa Cruz, CA, USA). CYP2E1 antibody was from Milipore (Temecula, California, USA). Chemiluminescence (ECL) detection kit was from Pierce Biotechnology (Rockford, IL, USA). TRI reagent was from Invitrogen (Carlsbad, CA, USA). RNase-free DNase was from Promega Corporation (Madison, WI, USA). *In Situ* Cell Death Detection Kit (Roche, Cat. No. 11684817910). All other reagents were purchased from Sigma Chemical Co. (St. Louis, MO) if not otherwise stated.

### Animals and treatments

Adult male CD-1 mice (8–10 week-old, 32~34 g), adult female CD-1 mice (8–10 week-old, 28~30 g), immature (weanling) male CD-1 mice (3 week-old, 14~16 g) and immature (weanling) female CD-1 mice (3 week-old, 12~14 g) were purchased from Beijing Vital River (Beijing, China). The animals were allowed free access to food and water at all times and were maintained on a 12-h light/dark cycle in a controlled temperature (20–25 °C) and humidity (50 ± 5%) environment. Forty adult male mice, 40 adult female mice, 40 immature male mice and 40 immature female mice were divided into sixteen groups (ten mice each group). After a 12-h fast, all mice were intraperitoneally (i.p.) injected with a single dose of APAP (300 mg/kg). Mice were sacrificed at different time points (0, 1, 4 or 24 h) after APAP injection (10 mice each time point). Serum samples were collected for measurement of biochemical parameters. Liver samples were collected and frozen immediately in liquid nitrogen for measurement of GSH, antioxidant enzyme activities, real-time RT-PCR and immunoblot. Some liver samples were fixed in neutral-buffered formalin for histological examination and TUNEL assay. For survival rate test, 10 adult male mice, 10 weanling male mice, 10 adult female mice and 10 weanling female mice were i.p. injected with APAP (300 mg/kg). Animal death was observed until 7 d after APAP. This study was approved by the Association of Laboratory Animal Sciences and the Center for Laboratory Animal Sciences at Anhui Medical University (Permit Number: 14-0017). All procedures on animals followed the guidelines for humane treatment set by the Association of Laboratory Animal Sciences and the Center for Laboratory Animal Sciences at Anhui Medical University.

### Evaluation of liver injury

Serum alanine aminotransferase (ALT) was measured using commercially available assay kits according to manufacturer’s instructions. Liver tissues were fixed in 4% formalin and embedded in paraffin according to standard procedure. Paraffin embedded tissues were cut 5 mm thick and stained with hematoxylin and eosin (H & E) for morphological analysis. To quantify the extent of necrosis, the percentage of necrosis was estimated by measuring the necrotic area relative to the entire histological section. An analysis of the necrotic area was performed with NIH ImageJ software (http://rsb.info.nih.gov/ij/).

### Hepatic GSH measurement

Hepatic GSH and GSSG were measured by DTNB–GSSG reductase recycling assay as described by Anderson^41^ with some modifications. Hepatic GSH content was expressed as μmol g-1 liver.

### Immunoblot

Hepatic lysate was prepared by homogenizing 50 mg liver tissue in 300 μl lysis buffer (50 mM Tris-HCl, pH 7.4, 150 mM NaCl, 1 mM EDTA, 1% Triton X-100, 1% sodium deoxycholate, 0.1% sodium dodecylsylphate, 1 mM phenylmethylsulfonyl fluoride) supplemented with a cocktail of protease inhibitors (Roche). The concentration of protein was determined by the bicinchoninic acid (BCA) protein assay (Pierce, Rockford, IL, USA). For immunoblot, same amount of protein (40–80 μg) was separated electrophoretically by SDS-PAGE and transferred to a polyvinylidene fluoride membrane. The membranes were incubated for 2 h with following antibodies: RIP1, RIP3, JNK, pJNK and CYP2E1. Alpha-tubulin was used as a loading control. After washed in DPBS containing 0.05% Tween-20 for four times for 10 min each, the membranes were incubated with either goat anti-rabbit or goad anti-mouse IgG antibodies for 2 h. The membranes were washed for four times in DPBS containing 0.05% Tween-20 for 10 min each, followed by signal development using an ECL detection kit.

### Isolation of total RNA and real-time RT-PCR

Total RNA in liver tissue was extracted using TRI reagent. The purity of RNA was assessed according to the ratio of absorbance at 260 nm and 280 nm. RNase-free DNase-treated total RNA (1.0 μg) was reverse-transcribed with AMV. Real-time RT-PCR was performed with Light Cycler 480 SYBR Green I Kit (Roche Diagnostics GmbH, Manheim, Germany) using genetic-specific primers as listed in Table 1. The amplification reactions were carried out on a LightCycler 480 Instrument (Roche Diagnostics GmbH, Mannheim, Germany) with an initial hold step (95 °C for 5 min) and 50 cycles of a three-step PCR (95 °C for 15 sec, 60 °C for 15 sec, 72 °C for 30 sec). The comparative CT-method was used to determine the amount of target, normalized to an endogenous reference and relative to a calibrator (2-ΔΔCt) using the Lightcycler 480 software (Roche, version 1.5.0)^42^,^43^. All RT-PCR experiments were performed in triplicate.

### Hepatic GSH metabolic enzyme activities

Liver tissues were homogenized in 50 mM phosphate buffer (pH 7.4) and centrifuged at 3200 g for 20 min at 4 °C. Se-dependent glutathione peroxidase (GPX) activity was measured according to the method as described by others^44^. GPX catalyses the oxidation of GSH in the presence of hydrogen peroxides. Oxidized GSH is converted into the reduced form in the presence of GSH reductase (GR) and NADPH, while NADPH is oxidized to NADP. Reduction in the absorbance change per minute and by using the molar extinction coefficient of NADPH, hepatic Se-dependent GPX activity was calculated. Se-dependent GPX activity was expressed as U/g protein. Hepatic GR activity was analyzed following NADPH oxidation at 340 nm in the presence of GSSG. Hepatic GR activity was expressed as U/g protein. Glutathione S-transferase (GST) activity was determined using the method as described by others^45^. Briefly, GST activity was measured by determining the rate of conjugate formation between GSH and 1-chloro-2,4-nitrobenzene (CDNB) at 340 nm. Hepatic GST activity was expressed as U/mg protein. Change in absorbance is a linear function of enzyme concentration. All enzyme activities were determined at 25 °C. The concentration of protein was measured by the BCA assay (Pierce, Rockford, IL, USA).

### Terminal dUTP nick-end labeling (TUNEL) staining

For the detection of nuclear DNA strand breaks, paraffin-embedded sections were stained with the TUNEL technique using an *In Situ* Cell Death Detection Kit ccording to manufacturer’s protocols. Sections were counterstained with hematoxylin. TUNEL-positive cells were counted in twelve randomly selected fields from each slide at a magnification of ×200. The percentage of TUNEL-positive hepatocytes was analyzed in ten liver sections from ten different mice.

### Statistical Analysis

All quantified data were expressed as means ± SE at each point. ANOVA and the Student-Newmann-Keuls post hoc test were used to determine differences among different groups.

## Additional Information

**How to cite this article:** Lu, Y. *et al*. Immature mice are more susceptible than adult mice to acetaminophen-induced acute liver injury. *Sci. Rep.* **7**, 42736; doi: 10.1038/srep42736 (2017).

**Publisher's note:** Springer Nature remains neutral with regard to jurisdictional claims in published maps and institutional affiliations.

## Supplementary Material

Supplementary Information

## Figures and Tables

**Table 1:** Primers for real-time RT-PCR.

Gene	Primer Sequence	Length (bp)
*18S*	F: GTAACCCGTTGAACCCCATTR: CCATCCAATCGGTAGTAGCG	151
*Gr*	F: GGGATGCCTATGTGAGCCGCCR: TGACTTCCACCGTGGGCCGA	120
*Gpx*	F: GGTGGTGCTCGGTTTCCCGTR: AATTGGGCTCGAACCCGCCAC	113
*Gst*	F: CTGAGTACCCCTCTGTCTACGCAGCR: CAGCATTCGCATGGCCTCACAC	110
*Cyp2e1*	F: CCGACCTGTTCTTTGCAGGAR: GCTTGGCCCAATAACCCTGT	128
*Cyp3a11*	F: CCTGGGTGCTCCTAGCAATCR: GGCCCAGGAATTCCCTGTTT	91
*Cyp1a1*	F: GCCGATCGGAGGTCTTTCTCR: AGCGGGCGTGTTTTAAAGTC	120
*Ugt1a1*	F: TGGACGGACTGCCTTTAATCAR: GGCCATAAACTCGGCATTGT	130
