# Supplementary material for: Immature mice are more susceptible than adult mice to acetaminophen-induced acute liver injury
Source: Sci Rep. 2017 Feb 16;7:42736. doi: 10.1038/srep42736 (PMC5311972; doi:10.1038/srep42736)
Supplement: Supplementary Information [file srep42736-s1.pdf]

Supplementary information

Immature mice are more susceptible than adult mice to acetaminophen-induced acute liver injury

Yan Lu[1,2,3], Cheng Zhang[1], Yuan-Hua Chen[1,2], Hua Wang[1], Zhi-Hui Zhang[1], Xi Chen[4,*], De-Xiang Xu[1,2,*]

[1] Department of Toxicology, Anhui Medical University, Hefei, 230032, China;

[2] Anhui Provincial Key Laboratory of Population Health & Aristogenics, Anhui Medical University, Hefei, 230032, China;

[3] Second Affiliated Hospital, Anhui Medical University, Hefei 230601, China;

[4] First Affiliated Hospital, Anhui Medical University, Hefei 230022, China

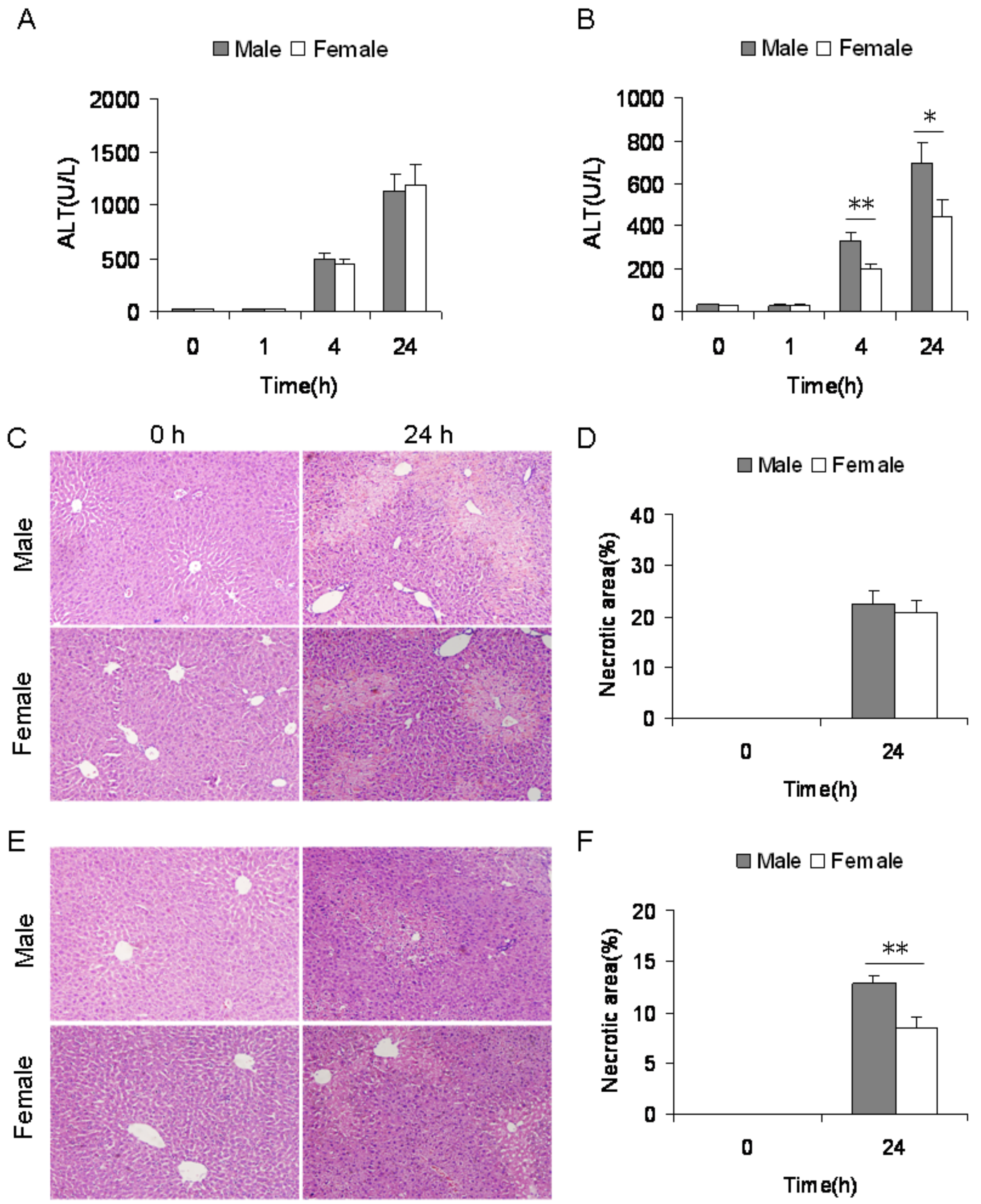

Supplementary Figure S1 Adult male mice are more susceptible than adult female mice to APAP-induced acute liver injury. Immature (weanling) and adult mice were i.p. injected with APAP (300mg/kg). Sera were collected at different time points (0, 1, 4 and 24 h) after APAP. (A) Serum ALT was analyzed in immature mice. (B) Serum ALT was analyzed in adult mice. (C) Representative photomicrographs of liver histology in immature mice. HE: 100×. (D) The percentage of necrotic area was analyzed in immature mice. (E) Representative photomicrographs of liver histology in adult mice. HE: 100×. (F) The percentage of necrotic area was analyzed in adult mice. All data were expressed as means ± SE (n=10). **P*<0.05, ***P*<0.01.

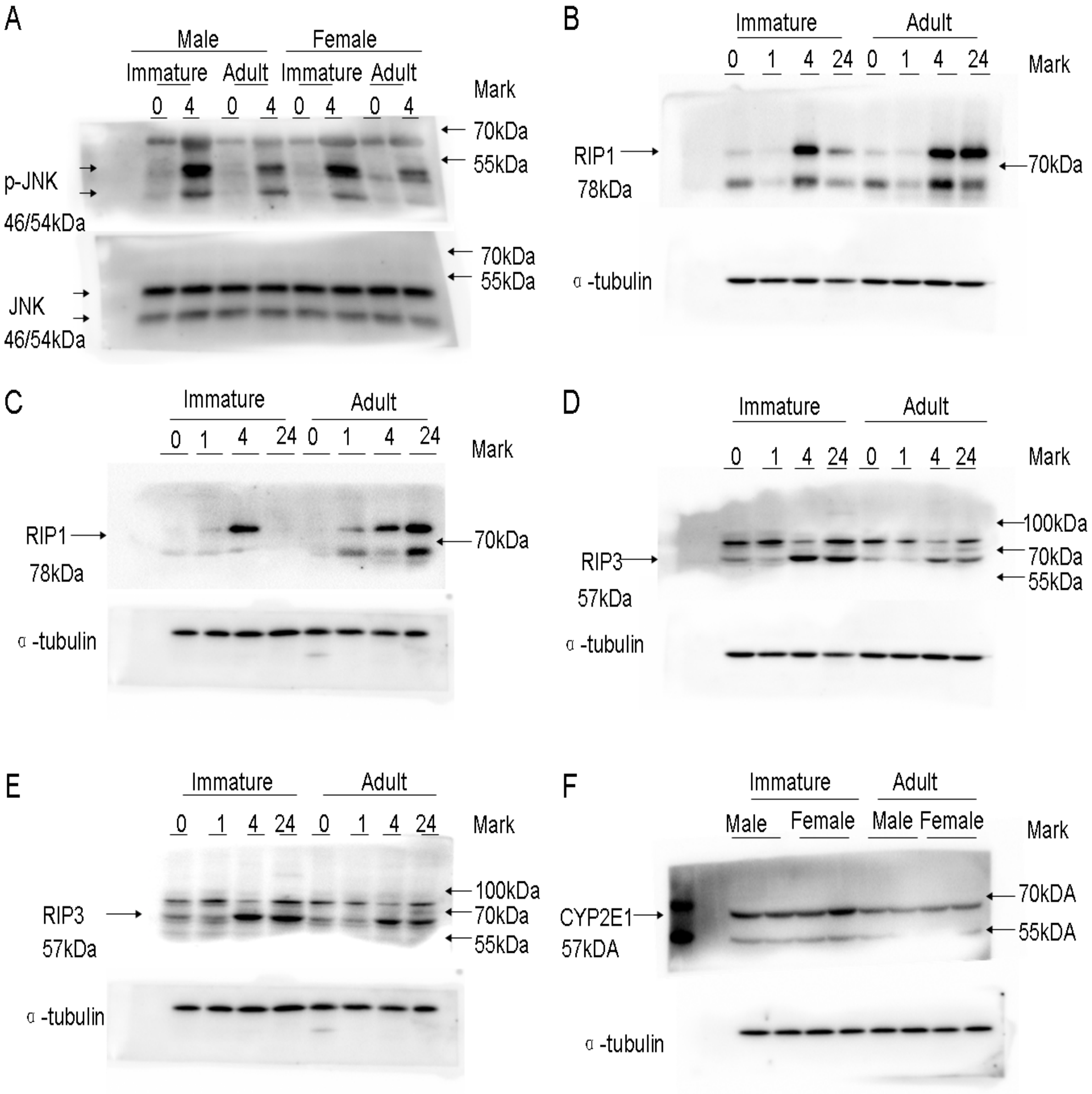

Supplementary Figure S2 Original images for Western blot. (A-E) Immature and adult mice were i.p. injected with APAP (300mg/kg). Liver samples were collected at different time points (0, 1, 4 and 24 h) after APAP. (A) A representative gel for pJNK (upper panel) and JNK (lower panel). The gel is processed into Figure 3A in the main figures. (B) A representative gel for RIP1 (upper panel) and α-tubulin (lower panel) in male mice. The gel is processed into Figure 4A in the main figures. (C) A representative gel for RIP1 (upper panel) and α-tubulin (lower panel) in female mice. The gel is processed into Figure 4C in the main figures. (D) A representative gel for RIP3 (upper panel) and α-tubulin (lower panel) in male mice. The gel is processed into Figure 4E in the main figures. (E) A representative gel for RIP3 (upper panel) and α-tubulin (lower panel) in female mice. The gel is processed into Figure 4G in the main figures. (F) Liver samples were collected from APAP-untreated immature and adult mice. A representative gel for CYP2E1 (upper panel) and α-tubulin (lower panel). The gel is processed into Figure 7I in the main figures.
